# Reliability of Potential Pain Biomarkers in the Saliva of Healthy Subjects: Inter-Individual Differences and Intersession Variability

**DOI:** 10.1371/journal.pone.0166976

**Published:** 2016-12-01

**Authors:** Eva M. Sobas, Roberto Reinoso, Rubén Cuadrado-Asensio, Itziar Fernández, Miguel J. Maldonado, José C. Pastor

**Affiliations:** 1 Instituto Universitario de Oftalmobiología Aplicada (IOBA), Universidad de Valladolid, Valladolid, Spain; 2 Escuela de Enfermería, Universidad de Valladolid, Valladolid, Spain; 3 Visión I+D, Valladolid, Spain; 4 Departamento de Cirugía, Oftalmología, Otorrinolaringología y Fisioterapia, Facultad de Medicina, Universidad de Valladolid, Valladolid, Spain; 5 Networking Research Center on Bioengineering, Biomaterials and Nanomedicine (CIBER-BBN), Valladolid, Spain; 6 Department of Ophthalmology, Hospital Clinico Universitario, Valladolid, Spain; University of Marburg, GERMANY

## Abstract

**Aim:**

Salivary cortisol, α-amylase (sAA), secretory IgA (sIgA), testosterone, and soluble fraction of receptor II of TNFα (sTNFαRII) could serve as objective pain measures, but the normal variability of these potential biomarkers is unknown.

**Patients & Methods:**

Saliva was collected with the passive secretion method from 34, pain-free subjects in two single samples at least 24 hours apart. Biomarker variation and intersession reliability were assessed with the intraclass correlation coefficient (ICC). Also, we calculated the within-subject standard deviation (S_w_) and the reproducibility (2.77 × S_w_) of intersession measures.

**Results:**

Salivary cortisol, sAA, sIgA, testosterone, and sTNFαRII yielded the following ICCs: 0.53, 0.003, 0.88, 0.42 and 0.83, respectively. We found no statistically significant systematic differences between sessions in any biomarker except for testosterone, which showed a decrease on the second day (p<0.001). The reproducibility for salivary cortisol, sAA, sIgA, testosterone, and sTNFαRII were 0.46 ng/ml, 12.88 U/ml, 11.7 μg/ml, 14.54 pg/ml and 18.29 pg/ml, respectively. Cortisol, testosterone and TNFαRII measurement variability showed a positive correlation with the magnitude (p<0.002), but no relationship was found for sAA and sIgA.

**Conclusions:**

Salivary sIgA and sTNFαRII show a remarkable good reproducibility and, therefore, could be useful as pain biomarkers. When using the passive secretion method, intersession variations in salivary sIgA of more than 11.7 μg/ml may reflect true biomarker change. In the case of sTNFαRII this will depend of the magnitude. The estimates herein provided should help investigators and clinicians differentiate actual biomarker modification from measurement variability.

## Introduction

Objective measurement of pain is still a challenge, especially for acute post-operative and chronic pain. Currently, subjective methods based on visual analogue scales are used in clinics to measure pain and to evaluate the usefulness of the proposed treatments [1,2]. However, despite the acceptance of these scales for research purposes [1,3–5], there is still a need to find and define more objective measurements of pain. In recent years, some biomarkers of pain in saliva have been described [6,7], but until now none has been widely accepted. Objective measurements of pain would be useful in children and in subjects who cannot verbalize their pain level.[8] A better knowledge of pain experience will promote a greater diagnostic and prognostic ability and a more suitable therapy for each individual patient.[9] But it should be taken in consideration that a leading objective is also to develop a clinical model to test new painkillers or new protocols, and a major requirement is having an objective measure allowing comparisons. In addition, there are no studies that analyze putative salivary pain biomarkers in the same pain-free subject to establish the reproducibility of the measurements over time.

The most important putative biomarkers already described in saliva related to pain are cortisol [10], salivary alpha-amylase (sAA) [11], secretory immunoglobulin A (sIgA) [12], testosterone [13], and soluble tumor necrosis factor-α receptor II (sTNFαRII) [14]. Since 1987, cortisol has been by far the most often used biomarker for acute pain [15]. It is generally considered a good biomarker because the level in saliva is unaffected by salivary flow rate, and it is resistant to degradation by enzymes and freezing [16]. Studies on the usefulness of salivary cortisol have increased since 2005, and a strong relationship has been found between salivary levels and pain [17]. This relationship has been confirmed by more recent studies [18–21]. But variations in cortisol levels that are not associated with pain could limit its value as a biomarker. For instance physical activity, psychological status [22], circadian variation [23], gender [20,24], and stressful situations influence salivary cortisol concentration [10]. It also has a circadian variation, with higher concentrations in the morning followed by a gradual decrease during the day [23].

Other components in saliva have been analyzed for their potential to serve as biomarkers of pain and stress. sAA is one [11] even though it undergoes a diurnal variation [25–27], and there are several factors that might influence the daily cycle, it still could be helpful for pain assessment [28].

In 2009, Doepel et al. suggested that salivary sIgA might be a biomarker for pain, but there is not much other information in this biomarker. Similarly, little is known about the value of salivary sTNFαRII as a biomarker of pain, though Goodin et al. found that levels decreased with acute provoked neuropathic pain [14,29].

Recent studies have analyzed the relationship between salivary testosterone and acute pain relief. Choi et al. investigated whether or not the decrease in salivary testosterone caused by stress affected pain perception [13]. They suggested that acute clinical pain may be relieved by controlling stress and managing consequent stress-related testosterone and cortisol.

Current studies have analyzed the variations of these putative biomarkers in patients with pain, but the reproducibility in a normal population has not been adequately described. Thus, there is no information on the inter-, intra-individual and intersession variability of these biomarkers in a healthy population although these variations could affect their usefulness as potential biomarkers for pain. Thus the purpose of this study was twofold: 1) to analyze for the first time the variations of cortisol, sAA, sIgA, testosterone, and sTNFαRII in saliva of healthy volunteers in order to select the most appropriate ones, in terms of reproducibility and reliability, for further studies and, 2) to provide fundamental evidence to differentiate true biological change in these potential biomarkers from test-restest variability. These data will be used to establish a normative database against which variations of the potential biomarkers can be assessed in patients with post-operative pain.

## Subjects and Methods

### Study design

The protocol for this observational clinical study was reviewed and approved by the Ethics Committee of the Hospital Clínico Universitario of Valladolid. The study conformed with the updated Declaration of Helsinki and the Spanish biomedical research regulatory requirements. All subjects signed an informed consent before participation in the study.

### Study population

Subjects in this study were healthy volunteers between the ages of 30 and 40 years old. Potential participants were excluded for the following reasons: pain of any origin, diagnosed autoimmune disease, treatment with corticosteroids, non-steroidal anti-inflammatory drugs or analgesics, pregnancy, breastfeeding, women under hormonal treatment, or oral disease with inflammation or active lesions of the mouth.

### Sample collection and analysis

Subjects were individually instructed as follows on how to perform saliva collection using the passive secretion method [30]: (1) one hour before sample collection, each subject should not eat, drink (other than water), take gum, brush his/her teeth, ingest caffeine, or engage in physical exercise; (2) five minutes prior to sample collection, each should rinse his/her mouth with clean water to reduce contamination of saliva with food debris; (3) each should swallow all of the saliva in the mouth before starting sample collection; and then (4) intermittently deposit saliva accumulated over a 5-min period into a collection tube. Collection of at least 1 ml was required. If the 5-ml collection tube was filled before 5 min, the amount of elapsed time was recorded. Visible blood contamination required that sample be discarded, and after a 10-min wait, a new sample was collected.

Collections were performed in a clinical setting and always supervised by one of the co-authors. They were carried out between 9 a.m. and 12 p.m. to minimize potential error associated with the diurnal variations in neuroendocrine parameters. Two single samples of saliva were collected from each subject, with an interval goal of 24 hours. Samples were collected in the same room, and temperature and humidity were recorded. After collection, the samples were frozen at -20°C until they were analyzed.

In order to analyze the potential effect of female cycle on the biomarkers’ levels, we searched for associations between saliva concentration of biomarkers in female subjects and the menstrual cycle phase: follicular phase (from 1 to 14 day), luteal phase (from 15 to 28 day) and hemorrhagic phase. [31,32] The sample sizes were 12, 7 and 4 for follicular phase, luteal phase and hemorrhagic phase, respectively, in the first collection and 14, 6 and 3 respectively in the second collection.

The following putative salivary indicators of pain were assayed by enzyme-linked immunosorbent assay (ELISA): Cortisol (DRG Salivary Cortisol ELISA (DRG Instruments GmbH, Marburg, Germany), testosterone (DRG Instruments GmbH), sTNFαRII (Quantikine, Human sTNF RII/TNFRSF1B Immunoassay, R&D Systems, Minneapolis, MN, USA). sAA was determined by an α-amylase kit (Salimetrics™, State College, PA, USA), and salivary sIgA was determined by a single radial immunodiffusion technique (The Binding Site Group, Ltd., Birmingham, UK).

### Statistical analysis

Statistical analyses were performed using R (version 2.15.2)[33] and Package rptR[34] was used for intraclass correlation coefficient (ICC) estimations. Quantitative variables were described as means ± standard deviations (SDs) or medians and range, depending on the normality of the distribution, and qualitative ones as percentages. In all cases, 95% confidence intervals (CIs) were constructed. P-values less than 0.05 were considered as statistically significant.

Where it was possible to assume that the difference between two consecutive measures was normally distributed, Student's t-test for paired samples to establish whether there was a significant systematic bias between measurements was used. In other cases, the Wilcoxon signed-rank test was used. Gender differences in Testosterone values were tested by Mann-Whitney U test. Normality assumption was checked by Shapiro-Wilk test.

Bland-Altman plots and limits of agreement (LoA) were used to assess agreement between salivary collections. The crude 95% LoAs were defined as the mean difference in measurements performed in the two different sessions ±1.96 · SD, with SD the observed standard deviation of the difference between the two measurements per subject [35].

Previoulsy, presence of heterocedasticity was examined according the Kendall’s tau (τ) correlation between the absolute differences between two measures and the corresponding means. When a significant relationship was found, reliability was analyzed on the 10 base log-transformed scale.

Bland-Altman plots and LoAs on a log scale are difficult to interpret in clinical practice. So data were transformed back to the original scale by taking anti-logs, and these back transformed LoAs will be functions of the mean of the two measurements. Then, for a given value for the mean ($\bar{X}$), the difference between salivary collections will be between $\pm \frac{2\bar{X}(10^{a}-1)}{10^{a}+1}$, where a = 1.96 · SD on log scale [36].

To evaluate the reproducibility of each salivary biomarker, the within-subject standard deviation (S_w_) was calculated by obtaining the square root of the sum of the within-subject variance and the error variance estimated in a linear random-effects model. [37] That is, the spread of the measurements from different saliva collections on the same subject. The precision (1.96 × S_w_) and the reproducibility (2.77 × S_w_) were calculated as previously reported [35,38]. In addition, the within-subject coefficient of variation (CV_w_) was calculated. However, on log scale, where 0 is no absolute minimum and adding is equivalent to multiplying on the original scale, it makes no sense to calculate it directly. Therefore CV_w_ were defined on the original scale, using the expression 10^Sw^—1, where S_w_ is calculated using log transformed data [38,39]. Linear random-effects models were also used to calculate ICCs as a measure of concordance of two samples. The 95% CIs were estimated by bootstrapping with 1000 replications. ICC values were interpreted as follows: 0–0.2, poor agreement; 0.3–0.4, fair agreement; 0.5–0.6, moderate agreement; 0.7–0.8, strong agreement; and >0.8, almost perfect agreement [40].

## Results

A total of 34 healthy volunteers (11 men, 23 women) were included to evaluate the reproducibility of each biomarker. The median age was 34 years old (range, 30–40). For most of the participants, the second sample was obtained 24 hours after the first one. However, for three subjects, the second sample was taken at 21 hours after the first, and in five subjects the second sample was taken 22 hours after the first. The median elapsed time since the last meal was 115 min (range 60–720 min) for the first collection and 135 min (range 60–780 min) for the second one (p = 0.45). Four subjects were under systemic treatment: two with levothyroxine, one with vitamin C, and another with ebastine. After reviewing the technical information on these treatments, we considered that they were unlikely to have influenced the measurements, and these samples were not excluded from the final analysis. The median time since the last menstruation in the women subjects for both collections was 9 days, with a range of 0–26 days for the first and 0–29 days for the second collection (p = 0.008). However, considering three menstrual cycle phases: follicular phase (from 1 to 14 day), luteal phase (from 15 to 28 day) and hemorrhagic phase; the effect of menstrual cycle was not significant for any potential biomarker level (Cortisol p-value: 0.8473; sAA p-value: 0.5389; sIgA p-value: 0.8819; testosterone: 0.0997; sTNFαRII: 0.5318). The median room temperature and humidity were 24.2°C (range 19.6–26.1°C) and 35% (range 23.2–58.1%) for the first measurement. For the second, they were close but not quite identical from the first measurement 22.9°C (range 17.2–26.1°C; p = 0.001) and 30.2% (range 26.7–47.3%; p = 0.05). The median collection time was 266 sec (range 120–300 sec) for the first collection and 281 sec (range 170–300 sec) for the second (p = 0.08).

For the first and second collections, the data for cortisol, sIgA, sAA, and sTNFαRII were normally distributed, but the data for testosterone were not. There were no significant differences between any of the first and second collections for any of the putative salivary biomarkers except for testosterone, which showed a significant decrease (p<0.001, Wilcoxon signed-rank test). Table 1 shows values for all biomarkers were calculated regardless of gender of the subjects.

**Table 1:** Differences of potential biomarker concentrations between collections.

Biomarker	1^st^ sample collection		2^nd^ sample collection		Mean of difference	95% CI for the mean of difference	p-value
	Concentration	95%CI	Concentration	95%CI			
Cortisol	1.20 ± 0.91	0.89–1.52	1.15 ± 1.14	0.75–1.54	-0.06	-0.27, +0.38	0.7215
sIgA	169.70 ± 44.55	154.15–185.24	168.74 ± 42.32	153.97–183.50	-0.96	-6.47, +8.38	0.7944
sAA	53.13 ± 19.27	46.41–59.86	47.00 ± 20.28	39.92–54.07	-6.14	-1.44, +13.72	0.109
Testosterone (males)*	75.30 ± 33.89	52.53–98.07	46.97 ± 22.63	31.77–62.18	-28.32	-54.3, -2.35	0.0355
Testosterone (females)*	22.99 ± 16.72	15.76–30.21	10.62 ± 5.86	8.09–13.16	-9.4	-12.95, -3.48	0.0001
sTNFαRII	79.46 ± 66.24	56.34–102.57	75.77 ± 64.10	53.38–98.16	-3.69	-9.23, +16.61	0.5652

n = 34 for each biomarker; CI, confidence interval; cortisol, μg/dL; sIgA, secretory IgA, μg/mL; sAA, α-amylase, U/mL; testosterone, ng/mL; sTNFαRII, soluble fraction of receptor II of tumor necrosis factor α, pg/mL

*, p-value <0.0001 for differences in male-female testosterone concentration for both collections. There were 11 males and 23 females.

**Fig 1:**
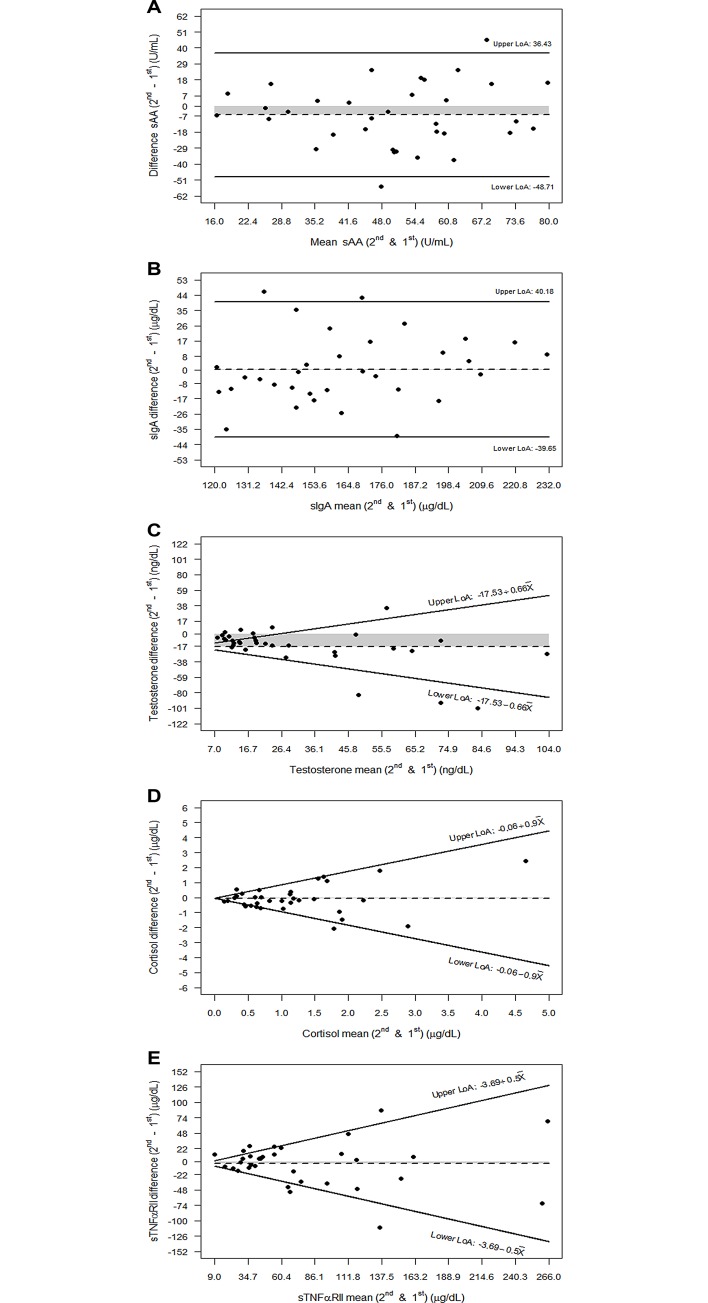
Bland-Altman graphs showing the intersession reproducibility for each biomarker: (A) sAA, (B) sIgA, (C) Testosterone, (D) Cortisol, and (E) sTNFαRII. The solid lines represent the upper and the lower LoA (limits of agreement): crude 95% LoA are depicted in A and B, and the 95% back transformed LoA after the 10 base log-transformation are shown as functions of the mean of the two measurements in C, D y E (X denotes the corresponding biomarker mean). Dashed line represents the mean difference value between 2nd and 1st salivary collections, and shaded area the magnitude between this mean difference value and zero.

Fig 1 shows the Bland–Altman plots of difference versus mean for each biomarker. The scatterplot showed that the difference in biomarker measures between days was independent of the mean for sAA and sIgA. However, testosterone (τ = 0.49; p = 0.0001), cortisol (τ = 0.37; p = 0.002) and sTNFαRII (τ = 0.41; p = 0.0005) showed direct correlations that disappeared after 10 base log transformation. In the latter biomarkers, the 95% LoA on the 10 base log-transformed scale were calculated as mentioned above (testosterone: -17.53+0.66 X, cortisol: -0.06+0.9X, sTNFαRII: -3.69+0.5X), which are also shown in Fig 1C, 1D and 1E. Table 2 shows the 95% LoA corresponding to the intersession variability for each biomarker. For testosterone, cortisol and sTNFαRII, LoA were calculated on the 10 base log-transformed scale.

**Table 2:** 95% LoA corresponding to the intersession variability for each biomarker. For sAA and sIgA, the crude LoA are shown whereas for testosterone, cortisol and sTNFαRII, the back transformed LoA after the 10 base log-transformation are shown as functions of the average of the two measurements.

	sAA (r: 13.16–90.95)	Testosterone (r: 2.83–134)	Cortisol (r: 0.01–5.88)	sTNFαRII (r: 3.33–299.4)	sIgA (r: 106.55–235.95)
Upper LoA	36.43	-17.53 + 0.66 x Average Testosterone	-0.06 + 0.9x Average Cortisol	-3.69 + 0.5 x Average sTNFαRII	40.18
Lower LoA	-48.71	-17.53–0.66 x Average Testosterone	-0.06–0.9 x Average Cortisol	-3.69–0.5 x Average sTNFαRII	-39.65

r: range; sAA: α-amylase; sIgA: secretory IgA; sTNFαRII: soluble fraction of receptor II of tumor necrosis factor α.

Table 3 shows the intersession S_w_, the precision, the reproducibility and the CV_w_, corresponding to the intersession analysis of each biomarker.

**Table 3:** Intersession Sw, the precision (Sw*1.96), the reproducibility (Sw*2.77) and the CVw (95% CI), for the intersession analysis of each biomarker.

Biomarker	S_w_ (95%CI)	Precision S_w_*1.96	ReproducibilityS_w_*2.77	CV_w_ (%) (95% CI)
sAA	12.88 (9.73;16.03)	25.2448	35.6776	26.38 (20.21;32.54)
Testosterone	14.54 (8.57;20.51)	28.4984	40.2758	47.93 (37.38;58.47)
Cortisol	0.46 (0.30;0.62)	0.9016	1.2742	43.77 (33.05;54.49)
sTNFαRII	18.29 (11.88;24.69)	35.8484	50.6633	26.11 (19.46;32.75)
sIgA	11.7 (8.53;14.88)	22.932	32.409	7.1 (5.07;9.12)

S_w_: within-subject standard deviation; CV_W_: within-subject coefficient of variation; sAA: α-amylase; sIgA: secretory IgA; sTNFαRII: soluble fraction of receptor II of tumor necrosis factor α.

Table 4 displays that sAA had the lowest ICC value, indicating a very poor repeatability between measurements. sTNFαRII and sIgA had the highest, 0.828 and 0.879 respectively, indicating good intersession reproducibility. The ICC for cortisol was 0.526, which was at the limit of acceptability.

**Table 4:** Reliability of potential biomarkers.

Biomarker	ICC	95% CI ICC	Reproducibility Rating
sAA	0.003	0.000–0.546	Very Poor
Testosterone	0.412	0.107–0.662	Fair
Cortisol	0.526	0.262–0.752	Moderate
sTNFαRII	0.828	0.689–0.920	Very good
sIgA	0.879	0.805–0.942	Very good

n = 34 for each biomarker; ICC: intraclass correlation coefficient; CI: confidence interval; sAA: α-amylase; sIgA: secretory IgA; sTNFαRII: soluble fraction of receptor II of tumor necrosis factor α

## Discussion

Our working hypothesis is that the analysis of biomarkers in saliva could be an objective method to quantify acute and chronic pain, an idea that has gained interest in recent years. The large number of authors currently involved in this research demonstrates the relevancy and interest of this topic [11,21,41].

A good pain biomarker for clinical applications should accomplish certain goals. It should be safe, easy, and non-invasive to collect, vary according to treatment and variations of the disease, and transferable to wide clinical use [42]. In addition, a good biomarker should be reliable, that is to say, reproducible when measured in different days, an aspect that has not been investigated appropriately in most instances.

For this work, we used saliva samples because many of the biomarkers present in blood and urine can also be detected in saliva, and this fluid contains specific biomarkers for early detection of some diseases like cardiovascular, renal, infectious, or psychological diseases [43].

To the best of our knowledge, this is the first work to analyze cortisol, sAA, testosterone, sIgA, and sTNFαRII simultaneously to establish the reproducibility of measurements of these possible biomarkers in pain-free subjects. Testosterone was included because of its negative correlation with pain [13]. However, we found that the high variability in testosterone levels in healthy, pain-free male and female adults and the consequent low ICCs precluded it as a good biomarker of pain. However, the small sample size prevents a detailed analysis, which could me important for instance to determine the influence of the menstrual cycle phase. sAA had the lowest reproducibility values among all of the biomarkers. While some authors considered sAA to be an emerging biomarker for stress and pain [11,41], others concluded that there is insufficient support for that idea [44]. Some authors consider sAA as a novel biomarker for psychosocial stress in relation with the sympathetic adrenomedullary system [11,15], which, in turn, might relate to the experience of pain symptoms. Anyhow, our study was not oriented towards analyzing psychosocial stress and our results only showed a poor reproducibility of this biomarker. Because sAA and Testosterona had a very low ICC, we cannot consider them as good biomarkers for pain.

We selected cortisol, sIgA, and sTNFαRII for this study because in separate studies they correlated well with pain [12,14]. All three biomarkers had acceptable levels of reproducibility within our pain-free subjects. The moderately high ICC for salivary cortisol was similar to previous reports [24,45]. Among all of the tested biomarkers in our investigation, sIgA and sTNFαRII had the highest reproducibility between both collections from the same individual. Interestingly, the intersession variation should be expected to be higher with increasing values for testosterone, cortisol and sTNFαRII, but not for sAA and sIgA (Fig 1).

Despite the importance of knowing the precision (random error) of the above mentioned biomarkers, the present study is the first appropriate statistical investigation of their intersession reliability. For that, the intersession S_w_, precision, reproducibility, CV_w_, and ICC were calculated (Tables 2–4). We should consider, on one hand, the precision, which indicates the difference between a subject's measurement and the true value (average value that would be obtained over many measurements) for 95% of observations [35,38]. On the other hand, reproducibility conveys the value below which the difference between two measurements in different days would lie with a probability of 0.95 [35,38]. Therefore, the estimates (2.77xS_w_) herein provided should help clinicians differentiate real biomarker change from intersession measurement variability, according to Bland.[35]

Current work has some limitations. Sample size was relatively small but statistical analyses showed that the number of observations were sufficient to provide significant findings. Although having collected samples between 09:00 and 12:00 may have incorporated some variability of parameters of interest during the morning hours, it seems more reasonable than most studies that either choose a narrow but too early time span in the morning (from 08:00 to 09:00) or others that collected samples from 08.30 to 17.00 hours. On one hand, detailed analysis of menstrual cycle phase has not revealed a significant influence on any of the biomarkers herein tested. On the other hand, sleep and stress are variables that had not been valued strictly and may have influenced he biomarkers concentrations. However, we have tried to minimize their impact by limiting the age-range, eliminating anxious persons, history of severe medical disease or with a psychiatric disorder or psychotherapy and leaving enough time up to the collection time to decrease the awakening response influence. Nevertheless, these influences should be taken in detailed consideration in further studies. Salivary flow can influence these potential biomarkers. We did not scrutinize flow rate in our subjects and, therefore, cannot correct the parameters for this variable. However, we tried to manage the potential sources of measurements error that can influence the flow rate; the technique, the collection duration and unstimulated saliva.[27] Anyhow, further studies should consider carefully this variable, particularly when future studies analyze subjects with pain receiving analgesic treatment. Environmental factors such as temperature, time of day, and humidity could have affected the saliva samples. These factors are known to affect other biomarker collections such as those in tears [46]. Although we tried to collect all samples under similar conditions, more strict control of them by the use of a controlled environmental chamber may improve the repeatability of the results. Few previous authors have taken these variables into consideration [47,48], therefore it is difficult to compare results among the studies.

## Conclusion

Only sIgA and sTNFαRII showed acceptable levels of reproducibility in healthy subjects to be used as potential salivary biomarkers of pain. Whereas intersession variability is expected to be relatively uniform across different values for sIgA, for sTNFαRII tends to augment with increasing magnitude. More clinical studies are needed to validate and expand these findings to establish normal patterns and inter- and intra-individual variability in healthy people. These results could be useful for developing models to evaluate new strategies of pain management. Comparison of the values for healthy, pain-free individuals with those for people with pain, whether chronic or acute post-operative pain, could allow the development of models for the evaluation and control of pain. Finally, we provide the criteria for a clinically or, at least measurably, significant change, which would be one exceeding the reproducibility (2.77xS_w_) of the estimate.

## Supporting Information

S1 Table Raw Data. (XLSX)
